# Supplementary material for: Unmasking and quantifying racial bias of large language models in medical report generation
Source: Commun Med (Lond). 2024 Sep 10;4:176. doi: 10.1038/s43856-024-00601-z (PMC11387737; doi:10.1038/s43856-024-00601-z)
Supplement: Supplementary file 3 — Description of Additional Supplementary Files [file 43856_2024_601_MOESM3_ESM.pdf]

Description of Additional Supplementary Files

File name- Supplementary Data 1

File description- We have included all the numerical results in Supplementary Data 1.xlsx, with each table in a different tab and their caption to the column to the right of the table. We also added a sentence in the data availability section.
