# Supplementary material for: Unmasking and quantifying racial bias of large language models in medical report generation
Source: Commun Med (Lond). 2024 Sep 10;4:176. doi: 10.1038/s43856-024-00601-z (PMC11387737; doi:10.1038/s43856-024-00601-z)
Supplement: Supplementary file 4 — Supplementary Data 1 [file 43856_2024_601_MOESM4_ESM.pdf]

	percentage	total	engine	z-test
White v. Black	0.585	1	GPT-3.5-turbo	0.000966848
White v. Asian	0.635	1	GPT-3.5-turbo	3.79791E-08
White v. Hispanic	0.575	1	GPT-3.5-turbo	0.001935206
Black v. Asian	0.57	1	GPT-3.5-turbo	0.006933948
Black v. Hispanic	0.53	1	GPT-3.5-turbo	0.271332122
Asian v. Hispanic	0.465	1	GPT-3.5-turbo	0.271332122

Supplementary data: GPT-3.5-turbo's projected hospitalization duration comparisons across different races.

	percentage	total	engine	z-test
White v. Black	0.59	1	GPT-3.5-turbo	0.000465258
White v. Asian	0.605	1	GPT-3.5-turbo	4.13E-05
White v. Hispanic	0.57	1	GPT-3.5-turbo	0.006933948
Black v. Asian	0.595	1	GPT-3.5-turbo	0.000215599
Black v. Hispanic	0.57	1	GPT-3.5-turbo	0.006933948
Asian v. Hispanic	0.475	1	GPT-3.5-turbo	0.368120251

Supplementary data: GPT-3.5-turbo's projected cost comparisons across races.

	percentage	total	engine	z-test
White v. Black	0.532338	1	GPT-4	0.231302188
White v. Asian	0.562189	1	GPT-4	0.016664731
White v. Hispanic	0.552239	1	GPT-4	0.046040843
Black v. Asian	0.517413	1	GPT-4	0.549502622
Black v. Hispanic	0.487562	1	GPT-4	0.689890453
Asian v. Hispanic	0.502488	1	GPT-4	1

Supplementary data: GPT-4's projected hospitalization comparisons across different races.

	percentage	total	engine	z-test
White v. Black	0.522388	1	GPT-4	0.318517344
White v. Asian	0.522388	1	GPT-4	0.424866156
White v. Hispanic	0.537313	1	GPT-4	0.110485458
Black v. Asian	0.477612	1	GPT-4	0.424866156
Black v. Hispanic	0.507463	1	GPT-4	0.841870183
Asian v. Hispanic	0.507463	1	GPT-4	0.689890453

Supplementary data: GPT-4's projected cost duration comparisons across races.

	Cost	Hospitalization
GPT-4	664	848
GPT-3.5-turbo	375	407

Supplementary data: Rate of inconclusive cost and hospitalization predictions by both models.

Model	Race	Deceased prediction rate	bootstrap_error
GPT-3.5-turbo	White	56.537753	2.116319
GPT-3.5-turbo	Black	62.246777	2.091599
GPT-3.5-turbo	Asian	58.747698	2.103239
GPT-3.5-turbo	Hispanic	59.668508	2.149171
GPT-4	White	32.780847	2.003839
GPT-4	Black	33.149171	2.003333
GPT-4	Asian	29.650092	1.956698
GPT-4	Hispanic	30.38674	1.981607

Supplementary data: Accuracy comparison in patient outcome predictions based on deceased patient reports by the two models.
