# Supplementary material for: Unmasking and quantifying racial bias of large language models in medical report generation
Source: Commun Med (Lond). 2024 Sep 10;4:176. doi: 10.1038/s43856-024-00601-z (PMC11387737; doi:10.1038/s43856-024-00601-z)
Supplement: Supplementary file 5 — Reporting Summary [file 43856_2024_601_MOESM5_ESM.pdf]

nature portfolio

Corresponding author(s): Zhiyong Lu

Last updated by author(s): Apr 17, 2024

Reporting Summary

Nature Portfolio wishes to improve the reproducibility of the work that we publish. This form provides structure for consistency and transparency in reporting. For further information on Nature Portfolio policies, see our Editorial Policies and the Editorial Policy Checklist.

Statistics

For all statistical analyses, confirm that the following items are present in the figure legend, table legend, main text, or Methods section.

n/a	Confirmed	
☐	☒	The exact sample size (*n*) for each experimental group/condition, given as a discrete number and unit of measurement
☐	☒	A statement on whether measurements were taken from distinct samples or whether the same sample was measured repeatedly
☐	☒	The statistical test(s) used AND whether they are one- or two-sided<br>*Only common tests should be described solely by name; describe more complex techniques in the Methods section.*
☒	☐	A description of all covariates tested
☒	☐	A description of any assumptions or corrections, such as tests of normality and adjustment for multiple comparisons
☐	☒	A full description of the statistical parameters including central tendency (e.g. means) or other basic estimates (e.g. regression coefficient) AND variation (e.g. standard deviation) or associated estimates of uncertainty (e.g. confidence intervals)
☐	☒	For null hypothesis testing, the test statistic (e.g. *F*, *t*, *r*) with confidence intervals, effect sizes, degrees of freedom and *P* value noted<br>*Give P values as exact values whenever suitable.*
☒	☐	For Bayesian analysis, information on the choice of priors and Markov chain Monte Carlo settings
☒	☐	For hierarchical and complex designs, identification of the appropriate level for tests and full reporting of outcomes
☒	☐	Estimates of effect sizes (e.g. Cohen's *d*, Pearson's *r*), indicating how they were calculated

Our web collection on statistics for biologists contains articles on many of the points above.

Software and code

Policy information about availability of computer code

Data collection	The patient notes used in this work is publicly available. For collecting large language model outputs, this work used custom code that makes API request to GPT models through Azure, all information needed to reproduce the code is detailed in the methods section.
Data analysis	We used python packages including pandas, seaborn, scipy, statsmodels, matplotlib and numpy for analysing and visualizing the collected data. All packages are publicly available.

For manuscripts utilizing custom algorithms or software that are central to the research but not yet described in published literature, software must be made available to editors and reviewers. We strongly encourage code deposition in a community repository (e.g. GitHub). See the Nature Portfolio guidelines for submitting code & software for further information.

Data

Policy information about availability of data

All manuscripts must include a data availability statement. This statement should provide the following information, where applicable:

- Accession codes, unique identifiers, or web links for publicly available datasets
- A description of any restrictions on data availability
- For clinical datasets or third party data, please ensure that the statement adheres to our policy

The patient notes used in this work is publicly available at https://huggingface.co/datasets/zhengyun21/PMC-Patients.

Human research participants

Policy information about studies involving human research participants and Sex and Gender in Research.

Reporting on sex and gender	N/A
Population characteristics	N/A
Recruitment	N/A
Ethics oversight	N/A

Note that full information on the approval of the study protocol must also be provided in the manuscript.

Field-specific reporting

Please select the one below that is the best fit for your research. If you are not sure, read the appropriate sections before making your selection.

☐ Life sciences ☒ Behavioural & social sciences ☐ Ecological, evolutionary & environmental sciences

For a reference copy of the document with all sections, see nature.com/documents/nr-reporting-summary-flat.pdf

Behavioural & social sciences study design

All studies must disclose on these points even when the disclosure is negative.

Study description	In this study, we provide qualitative and quantitative analysis of racial bias in GPT models with respect to 4 races.
Research sample	In total, this study uses 400 PubMed articles as source input, and generated 36,800 generated responses that covers different bias evaluations with respect to White, Black, Asian and Hispanic race. These responses are specifically designed to assess biases across four racial groups: White, Black, Asian, and Hispanic. This selection spans a spectrum from data-rich to underrepresented categories, ensuring a broad coverage in the evaluation of racial bias.
Sampling strategy	We sample from the last 4% in chronological order of the Pubmed articles included in PMC-Patients (a large dataset of Pubmed articles) to ensure that the selected cases are not seen by the model during training.
Data collection	Data are collected with GPT-3.5-turbo version 0301 and GPT-4 version 0613 using version 2023-03-15-preview Azure API.
Timing	Data are generated from August 2023 to November 2023.
Data exclusions	N/A
Non-participation	N/A
Randomization	N/A

Reporting for specific materials, systems and methods

We require information from authors about some types of materials, experimental systems and methods used in many studies. Here, indicate whether each material, system or method listed is relevant to your study. If you are not sure if a list item applies to your research, read the appropriate section before selecting a response.

Materials & experimental systems

n/a	Involved in the study
☒	☐ Antibodies
☒	☐ Eukaryotic cell lines
☒	☐ Palaeontology and archaeology
☒	☐ Animals and other organisms
☒	☐ Clinical data
☒	☐ Dual use research of concern

Methods

n/a	Involved in the study
☒	☐ ChIP-seq
☒	☐ Flow cytometry
☒	☐ MRI-based neuroimaging
